# Nipple–Areola Complex Reconstruction

**DOI:** 10.3390/medicina56060296

**Published:** 2020-06-16

**Authors:** Andrea Sisti

**Affiliations:** Department of Plastic Surgery, Cleveland Clinic, Cleveland, OH 44195, USA; asisti6@gmail.com

**Keywords:** nipple reconstruction, areola reconstruction, breast reconstruction, nipple–areola reconstruction, local flap, skin graft

## Abstract

The reconstruction of the nipple–areola complex is the last step in the breast reconstruction process. Several techniques have been described over the years. The aim of this review is to provide clarity on the currently available reconstructive options.

## 1. Introduction

Whenever possible, the surgeon spares the nipple areola complex (NAC) during breast demolition, through skin-sparing mastectomy or through nipple-sparing mastectomy [1]. In general, a tumor to nipple distance measured preoperatively by a digital mammogram of 2.5 cm or more is safe for NAC preservation in patients considered for breast conservation therapy [2].

When this is not possible, the areola–nipple complex is surgically removed together with the cancerous breast tissue and the nipple–areola complex is reconstructed afterward or in the same surgical session [3].

The reconstruction of the nipple–areola complex represents the final step of the breast reconstruction journey and it is generally performed four to six months after breast reconstruction [4,5,6,7,8]. The nipple–areola complex represents a very important anatomical part for the woman and the reconstruction has considerable aesthetic and psychological consequences [9,10,11].

Nevertheless, some women decline the reconstruction of the NAC after breast reconstruction [12]. Patients with later-stage cancer and a history of implant removal are less likely to have NAC reconstruction [12]. Satisfaction determinants are projection, color match, shape, size, texture, and position [13].

Several techniques have been proposed for the reconstruction [4,5,6,7,14,15,16,17,18]. Some authors have attempted to clarify the available surgical techniques for nipple–areola complex reconstruction. The first was Farhadi et al. [15] (Switzerland) in 2006, followed by Boccola et al. [14] (Australia) in 2010, Nimboriboonporn and Chuthapisith [17] (Thailand) in 2014 and Sisti et al. [5] (Italy) in 2016. In particular, Nimboriboonporn and Chuthapisith [17] proposed a first classification of nipple–areola reconstruction, according to the performed technique and to the type of material eventually grafted inside the neo-nipple. More recently (2018), Gougoutas et al. [16] published a quite comprehensive review on nipple–areola complex reconstruction.

It is important to distinguish between techniques used to reconstruct the nipple and techniques used to reconstruct the areola. Even though the nipple–areola is a complex, different techniques are performed to reconstruct the nipple and the areola. The literature on this topic is vast, but I will try my best to make it clear in this review.

## 2. Location of the Nipple and Areola Size

The location of the nipple on the reconstructed breast is a debated topic [19,20,21]. Pennisi et al. [20,21] proposed to place the new nipple 1/2 inch lateral to the mid-clavicular line on the sixth rib or interspace, while dr. Barnett [19] suggested to leave the decision about the new nipple–areola complex placement up to the patient.

Regarding the areola size, the choice is usually left to the patient if both the areolas have to be reconstructed, or the contralateral areola size is used as a model if only one areola needs to be reconstructed.

Laschuk et al. [22] recently proposed that the breast base width can be used to determine the ideal areolar size, using the areola: base width ratio of 0.29.

## 3. Nipple Reconstruction with Local Flap

The local flap is the most used technique for the reconstruction of the nipple [5,8,14,15,16,17]. This is a skin flap that usually includes the local skin and the superficial layer of the underlying subcutaneous tissue. 

In 1946, Berson [23] firstly described the use of a local skin flap for nipple reconstruction. Each surgeon uses the flap technique that is most familiar with, since the superiority of one technique over the others has not been demonstrated [5,24]. 

The most commonly used flaps are the arrow flap [25,26], the C-V flap [27] and the C-H flap [3]. 

Some techniques involve the projecting of the central area of the areola with purse string sutures, overgrafts, and buried grafts [28]. New design flaps are continuously described [29,30].

The main issue is maintaining the nipple’s projection [31] over time, since the new local flap tends to flatten. Sometimes the repetition of the same flap is necessary after the first surgery [32].

## 4. Use of Autologous/Allogenic/Synthetic Grafts to Improve Nipple Projection

To overcome the loss of projection, several material grafts have been proposed inside the new nipple: autologous tissue (fat [33], cartilage [25,34,35,36,37,38], derma [39]), allogeneic tissue (acellular dermal matrix [40,41], lyophilized allogeneic costal cartilage [42]), and synthetic materials (fillers [43,44,45] et al. [46,47]) can be grafted inside the nipple. Winocour et al. [18] have published an interesting review on this topic, concluding that the autologous tissue grafted inside the nipple has led to the best results.

More recently, Oliver et al. [46] focused their attention on allogeneic and alloplastic augmentation grafts in nipple–areola complex reconstruction in a systematic review, finding that the use of Ceratite (artificial bone) led to the highest complication rates [46].

Jankau et al. [48] proposed the use of a silicone rod inside the neo-nipple to enhance the projection over time, but the complications′ rate was high (10/30 patients developed flap necrosis followed by rod removal).

Tierney et al. [49] and Collins et al. [50] described the use of the Biodesign Nipple Reconstruction Cylinder (a rolled cylinder of extracellular matrix collagen derived from porcine small intestinal submucosa) inside the nipple reconstructed using skin flaps, with positive outcomes.

A tightly rolled dermal graft [51] might also be used for nipple reconstruction as well, in order to improve the long-term maintenance of nipple projection.

## 5. Areola Reconstruction Using Skin Graft

The reconstruction of the areola can be performed with a skin graft from hyper-chromic skin areas, like the inguinal (the inner thigh skin), the axillary region or the labia minora skin [52].

In 1949, Adams [53] described the first areola reconstruction using a full-thickness skin graft (FTSG) from the labium minora. This surgical operation is easy to perform and the healing process in both the donor and recipient sites is generally fast. The complication rate associated with the use of the skin graft for the areola reconstruction is low but higher when compared to the tattoo [5].

The main issue associated with the use of a FTSG for the areola reconstruction is the fading of the pigmentation over time. It is not uncommon to perform the skin graft again, one to two years after the initial graft.

## 6. Nipple Sharing Technique for Nipple Reconstruction

This technique can be performed only in case of unilateral breast reconstruction. It is a skin graft from the contralateral nipple [54]. The surgical procedure is well described in a video recently published by Gougoutas et al. [16]. The nipple is divided in half on a sagittal plane and then the harvested half nipple is grafted on a previously de-epithelized circular skin area on the opposite breast [16].

The breastfeeding functionality can be preserved using a nipple-sharing technique that does not damage the anatomic structure of the donor nipple for breastfeeding [55]. This technique was described by Sakai and Taneda and consists of harvesting tissue by the circumcision method of nipple reduction and grafting the tissue in a spiral configuration [55].

The surgeon can choose to use no sutures after graft removal and letting the donor nipple heal spontaneously, minimizing scarring preserving the natural appearance and good sensitivity of the donor nipple [56].

## 7. Tattooing of the Nipple–Areola Complex

The tattoo of the nipple–areola complex is commonly performed four to six months after the surgical reconstruction of the nipple [5]. Nevertheless, the use of local flaps for nipple reconstruction and medical tattooing of the NAC in one session has been described [57].

The procedure is easy to perform in an outpatient setting and can be performed by a non-medical professional as well. It is a safe procedure [5] and leads to a high level of satisfaction [58,59]. Sometimes, just the areola is tattooed, for instance the nipple can be reconstructed using the nipple–sharing technique and the areola can be tattooed [60].

The tattoo can be made before the nipple reconstruction, but this may modify the final circular border of the areola after nipple reconstruction. However, the sequence of nipple reconstruction and tattooing has no significant effect on the projection of the reconstructed nipple [61].

Sasaki et al. [62] proposed four tips for tattooing procedures in nipple–areola complex reconstruction: blurring the areola margin, creating the illusion of the Montgomery glands (areolar bumps), adjusting the areola position to achieve symmetry and creating the illusion of the height of the nipple by using shading.

Some authors have proposed to have the tattoo done before surgery [5]. Furthermore, the reconstruction of the whole areola–nipple complex by 3-D tattoo is more and more widespread [59,63,64].

Post-tattooing complications are rare. Joseph et al. [65] recently reported a delayed hypersensitivity reaction around tattooed nipple areolar complexes in a 33-year-old female nonsmoker patient. Starnoni et al. [66] described a rare case of nipple–areolar complex partial necrosis following micropigmentation.

As for the FTSG, because of fading of the pigment, further tattooing may be required, and areolar color mismatch is another possible eventuality [52]. Allergic contact dermatitis of the breast secondary to pigment reaction related to the areola tattoo has also been described [67].

## 8. Other Techniques

External nipple–areola prosthetics made of silicone or other materials are another possible option for the reconstruction [68,69,70,71,72]. The use of internal nipple prosthesis [73] has been described as well, but is less common.

Dermoabrasion to re-create a nipple areola complex has been proposed by Cohen [74] in a black patient, resulting in a hyperpigmented area without the need for a skin graft. Furthermore, De Cholnoky [75] proposed the eversion of the navel to re-create the nipple when abdominal skin tissue is used to reconstruct the breast.

## 9. Projection

The loss of projection over time is the main issue after nipple reconstruction, even though the patient satisfaction and projection are not necessarily related, as observed by Jones and Erdmann [38].

Few et al. [31] observed a consistent 60% loss of intraoperative nipple projection in a study on 93 patients, with two years of follow-up. Lee et al. [61] found a mean loss of projection of 52.5–55.1% after six months.

In general, is advisable to build in the first place a nipple taller/bigger than the contralateral, since about 50% of projection is expected [5,17,61]. The local skin flap’s thickness influences the neo-nipple projection, as observed by Ishii et al. [76].

The sequence of nipple reconstruction and tattooing does not affect the projection of the reconstructed nipple, as observed by Lee et al. [61] in a study on 394 reconstructed nipples. The use of graft augmentation showed a minor loss of nipple projection but might expose to an increased risk of complications [48].

## 10. Complications

In general, the incidence of complications is very low (0–11%) and the results in terms of patient satisfaction are remarkable [5,13,16,77].

The possible complications are nipple necrosis, tip loss, wound infection and wound breakdown [77]. Nipple necrosis is a rare event, due to the dual nature of the local flap. The local flap for nipple reconstruction is, in fact, a random skin flap that benefits also of imbibition from the underneath and surrounding skin areas (like skin grafts), given the very thin thickness of the skin flap itself. In the case of previous postmastectomy radiation, the reconstruction of the nipple is associated with an increased complication risk [77,78]. Implant-based breast reconstruction might be associated with higher rate of nipple reconstruction issues [77].

The local flap is the safest described technique for the nipple reconstruction [5]. The tattooing of the areola showed a lower number of complications compared to the areola reconstruction using a skin graft [5].

Fading after tattoo or after skin graft is common over time, therefore it is advisable to use a darker pigment for the tattoo or to choose a darker skin area as a place where to harvest the skin graft in the first place.

## 11. Conclusions

Nipple–areola complex reconstruction is a very important step in the breast reconstruction journey. A comprehensive understanding of the available options for reconstruction is of paramount importance for the plastic surgeon.
